# Patients’ perceptions of recovery following a 6-week exercise intervention for the treatment of patellofemoral pain: A mixed methods study

**DOI:** 10.4102/sajp.v75i1.684

**Published:** 2019-07-25

**Authors:** Dominique C. Leibbrandt, Quinette A. Louw

**Affiliations:** 1 Department of Health and Rehabilitation Sciences, Division of Physiotherapy, Stellenbosch University, Cape Town, South Africa

**Keywords:** anterior knee pain, rehabilitation, patients’ perspective, qualitative research, exercise intervention

## Abstract

**Background:**

Patellofemoral pain (PFP) is a common and complex condition. The diagnosis and causal mechanisms are not well understood and therefore the long-term prognosis tends to be poor. Exercise is currently the only evidence-based treatment strategy suggested to improve pain and function in the long term. However, no qualitative studies have been conducted to establish patients’ perceptions of recovery in the long term following an exercise intervention.

**Objectives:**

To measure self-reported recovery on a 7-point Likert scale in 31 participants with PFP 6 months after a 6-week physiotherapy intervention. To explore the subjective accounts of patients who received a physiotherapy intervention for PFP, regarding their expectations and perceptions of recovery.

**Method:**

Semi-structured exit interviews were conducted electronically 6 months after intervention to ascertain the patients’ perspectives on whether expectations of treatment were met, and factors that influenced their recovery experience.

**Results:**

Quantitative analysis of self-reported recovery on a 7-point Likert scale showed that 48.4% of participants felt that they were ‘recovered’. Qualitative analysis showed three main categories: expectations of treatment, perceptions of recovery and changes in functional abilities.

**Conclusion:**

Clinicians should address patients’ expectations of treatment and include the patients in decision-making regarding their treatment. Long-term follow-up is essential to ensure that treatment effects have been maintained, and this should include information about patients’ self-reported recovery.

**Clinical implications:**

This study suggests that patients’ expectations of treatment and perceptions of recovery from PFP may influence prognosis. Clinicians need to collaborate with patients and involve them in decision-making to achieve their goals. An individualised treatment approach is essential to adequately address patients’ experiences, priorities and beliefs.

## Introduction

### Background

Patellofemoral pain (PFP) is a common and disabling condition amongst adults and adolescents and may account for up to 17% of all knee-related complaints seen in primary healthcare clinics (Taunton et al. 2002).

Patellofemoral pain is troublesome as it impairs functional ability, tends to be persistent and, as a result, has a significant impact on time lost at work and in sports’ participation (Collins et al. 2013). In addition, the long-term prognosis is poor, as approximately 40% of patients presenting with the condition will experience an unfavourable self-reported recovery after 12 months despite receiving treatment (Van Linschoten et al. 2009). It has been estimated that only a third of all patients diagnosed with PFP are pain-free 1 year later (Rathleff et al. 2014). Exercise appears to be the only long-term recommended treatment, based on the 2016 PFP consensus statement (Crossley et al. 2016). However, there is limited and low-quality long-term evidence for the effect of exercise in the treatment of PFP (Witvrouw et al. 2005).

### Trends

The exact causes of PFP are unknown; however, recent evidence suggests that multiple factors such as physical, biological, psychological and social factors may contribute towards ongoing pain (Falla & Hodges 2017).

Therefore, individualised and multimodal treatment approaches are currently recommended (Crossley et al. 2016). To our knowledge only one qualitative study regarding PFP has been conducted (Smith et al. 2018). This study (Smith et al. 2018) used a qualitative method to investigate the experience of people living with PFP in the UK. The respondents identified how their pain had impacted their lives. Factors such as self-identity, confusion regarding causes of their pain, fear-avoidance and inappropriate coping strategies and fear of future damage were described by the participants. It was apparent that PFP resulted in a loss of physical ability in these patients and therefore had a significant impact on their lives. In patients with chronic musculoskeletal pain, the inability to continue with meaningful activities of daily living has been identified as a cause of anxiety (Roy 2004). Therefore, it is necessary to investigate how patients experience and respond to physiotherapy interventions in terms of regaining their functional abilities.

Qualitative research is valuable as it provides complex descriptive evidence of how participants experience a given research issue, in this case recovery after an exercise intervention for PFP. This information provides us with a deeper understanding of the evidence from the perspective of the participants experiencing the process (Lambert & Lambert 2012). This type of research is especially useful when used along with quantitative methods to better interpret the implications of the findings for the participants concerned (Mack et al. 2005). To our knowledge this is the first paper to qualitatively assess patients’ perceptions of recovery from PFP.

### Objectives

- To determine the long-term effect of an individualised exercise intervention on self-reported recovery in 31 participants who have been diagnosed with PFP.
- To explore the subjective accounts of participants who had received physiotherapy exercise intervention for PFP regarding their expectations and perceptions of recovery 6 months after the intervention.

### Contribution to field

It is necessary to ascertain whether patients feel that they have improved and that their needs and expectations of physiotherapy treatment have been met. This will enable researchers to tailor treatment plans to the individual as opposed to using a ‘one-size-fits all’ approach. This study has a novel individualised approach that combined quantitative and qualitative findings to provide insight about how patients responded to treatment.

This approach exemplifies the WHO’s policy framework for person-centred healthcare (2007). According to these guidelines, person-centred healthcare should be prioritised so that individuals, families and communities have access to a trusted healthcare system that meets their needs (Yardley et al. 2015). This approach promotes collaboration between individuals, clinicians and healthcare organisations to improve the quality and responsiveness of the provided healthcare. It serves to empower patients by including them in decision-making regarding their health.

## Research methods

### Research approach

A descriptive qualitative approach was used to identify how patients had experienced an intervention approach, by gathering extensive data from a small number of participants (Curry, Nembhard & Bradley 2009). The qualitative descriptive design is used to comprehensively summarise the experiences of the group of individuals in a straightforward logical manner using everyday terms (Lambert & Lambert 2012). Information was collected retrospectively by conducting semi-structured exit interviews at 6 months post-intervention.

### Setting

Our study was conducted at the Tygerberg Physiotherapy Clinic and 3D Movement Analysis Laboratory of Stellenbosch University, Cape Town, South Africa. This qualitative study was part of a larger quantitative study, wherein participants underwent motion analysis procedures and a clinical assessment at the Tygerberg 3D Movement Analysis Laboratory. They thereafter participated in a 6-week individualised exercise intervention based on their person-specific biomechanical contributing factors, which took place in the gym of the Tygerberg Physiotherapy Clinic (Leibbrandt & Louw 2019). The interventions were administered by one of two physiotherapists (M.M. and D.L.). Both physiotherapists were females in their late 20s with experience working in private sport injuries practices. The first author D.L. trained M.M. to administer the interventions according to the study guidelines. The exit interviews were conducted telephonically. The same two therapists who did the supervised treatment sessions (M.M. and D.L.) phoned the participants to conduct the follow-up interviews.

### Design

A descriptive mixed methods approach was used. This approach has been recognised as valuable as it allows the researcher to capitalise on the strengths of both approaches (Curry et al. 2009).

### Sample recruitment

For pragmatic reasons a purposive sampling method was used. Participants were initially recruited for a larger n of 1 design exercise intervention study (Leibbrandt & Louw 2019) and the same participants were followed up at 6 months post-intervention for the purpose of this study. Participants were recruited by placing advertisements in community, university and school-based newsletters. Adverts were also placed on social media (such as Facebook), targeting sports group and physiotherapy clinic pages. Adverts were displayed at the campus physiotherapy clinics, on noticeboards and on advertising screens on the medical campus of Tygerberg and the main campus of Stellenbosch University. The included participants were screened according to an evidence-based screening tool that had been developed specifically for the project (Leibbrandt & Louw 2017). This tool was developed to ensure standardised diagnosis and exclusion of other pathologies, based on an up-to-date evidence synthesis of systematic reviews. The included population comprised 31 participants between the ages of 14 and 40 with unilateral PFP, residing in the Cape Metropolitan Area.

### Procedure

Semi-structured exit interviews (Online Appendix 1) were conducted 6 months after the intervention period had ended. The follow-up interviews took no longer than 10 min to complete. The interviewer recorded the exact responses on a hard copy of the questionnaire for each participant. The responses were written down or recorded and typed immediately following the interview. Data collection through telephonic interviews, as an alternative to real-time personal interviews normally preferred in qualitative research, is acceptable, time efficient and economical (Opdenakker 2006). Using this approach, we were successful in obtaining completed interviews for all 31 of the included participants.

### Data analysis

Quantitative findings were summarised numerically in tables and then analysed descriptively. Results were expressed as means and standard deviations for continuous data or frequencies and percentages for categorical data. The primary quantitative outcome was long-term recovery on a 7-point Likert scale at 6-month follow-up (post-intervention period).

A thematic analysis approach was used to analyse the qualitative data. This is an independent approach within the descriptive qualitative methodologies commonly used to identify, analyse and report common themes within a set of data (Vaismoradi, Turunen & Bondas 2013). The first author (D.L.) typed the hand-written transcripts and then identified common themes in the set of interviews. The common themes were defined, checked and discussed by both authors (D.L. and Q.L.). The participants’ responses were then coded and exported into an Excel spreadsheet in order to identify categories that emerged within each theme. The spreadsheet can be seen in Online Appendix 2. The qualitative findings were reported by describing frequencies and proportions of responses that fit into certain categories. The three main themes for the qualitative outcomes were the patients’ expectations of physiotherapy at the time that participants volunteered for the study, patient perceptions on whether expectations had been met 6 months after the intervention, and the patients’ perspectives on the impact of the intervention on their ability to perform activities of daily living that they had identified as problematic or important.

### Ethical considerations

Ethical approval for the project was obtained from the Health Research Council of Stellenbosch University under ethics number N13/05/078. Protocol amendments were submitted annually. Informed consent was obtained from each participant prior to the commencement of the study. Informed assent was obtained from parents/guardians for participants under the age of 18 years.

## Results

### Sample description

Thirty-one participants (13 males, 18 females) with unilateral PFP (20 left-sided, 11 right-sided) were included in our study. The average age was 30(±8.4) (range 14-40), the mean height was 170.1(±10.4) cm and the mean weight was 77.5 (±25.7) kg. Twenty-five (80.6%) of the participants were active and participated in a sport or athletic activity regularly. The majority of the participants were working adults (74.2%); however, we also included six school-aged adolescents (19.4%) between the ages of 14 and 19 and two full-time university students (6.5%). The average duration of symptoms was 16.5 months and 68% of the participants had tried previous treatment such as massage, physiotherapy, biokinetics, taping, pain medication and strength training, as shown in Table 1.

**TABLE 1:** Sample description.

Pt ID	Affected leg (Left or Right)	Age (years)	Gender (Female or Male)	Duration of symptoms (months)	Previously sought treatment for condition (Yes or No)	Employed (Yes or No)	Active or sedentary?
P01	Left	38	Female	6	Yes	Yes	Active
P02	Right	18	Female	6	Yes	No	Sedentary
P03	Left	39	Female	3	No	Yes	Active
P04	Right	29	Male	3	No	Yes	Active
P05	Left	31	Male	12	No	Yes	Active
P06	Right	37	Male	24	No	Yes	Active
P07	Right	15	Female	48	Yes	No	Sedentary
P08	Left	40	Male	18	No	Yes	Active
P09	Right	18	Male	24	Yes	No	Sedentary
P10	Right	14	Female	18	Yes	No	Active
P11	Left	37	Female	6	No	Yes	Active
P12	Left	17	Female	60	Yes	No	Sedentary
P13	Right	14	Female	60	Yes	No	Active
P14	Left	39	Female	24	Yes	Yes	Active
P15	Left	31	Male	24	No	Yes	Active
P16	Right	40	Female	3	Yes	Yes	Active
P17	Right	40	Male	24	Yes	Yes	Active
P18	Left	25	Male	3	Yes	No	Active
P19	Left	24	Female	12	No	No	Active
P20	Right	29	Female	12	Yes	Yes	Active
P21	Left	34	Female	36	Yes	Yes	Active
P22	Left	33	Female	24	Yes	Yes	Active
P23	Left	31	Male	6	Yes	Yes	Sedentary
P24	Left	40	Female	6	Yes	Yes	Active
P25	Left	27	Male	4	No	Yes	Active
P26	Left	31	Female	12	Yes	Yes	Active
P27	Left	33	Male	84	Yes	Yes	Active
P28	Left	27	Female	48	No	Yes	Active
P29	Left	37	Female	3	Yes	Yes	Sedentary
P30	Left	36	Male	18	Yes	Yes	Active
P31	Left	32	Male	24	Yes	Yes	Active

*Source*: Leibbrandt, D. & Louw, Q., 2019, ‘The effect of an individualised functional retraining intervention on pain, function and biomechanics in participants with patellofemoral pain: A series of n of 1 trial’, *Journal of Physical Therapy Science* 31(1), 39–52. https://doi.org/10.1589/jpts.31.39

### Self-reported long-term recovery

**FIGURE 1:**
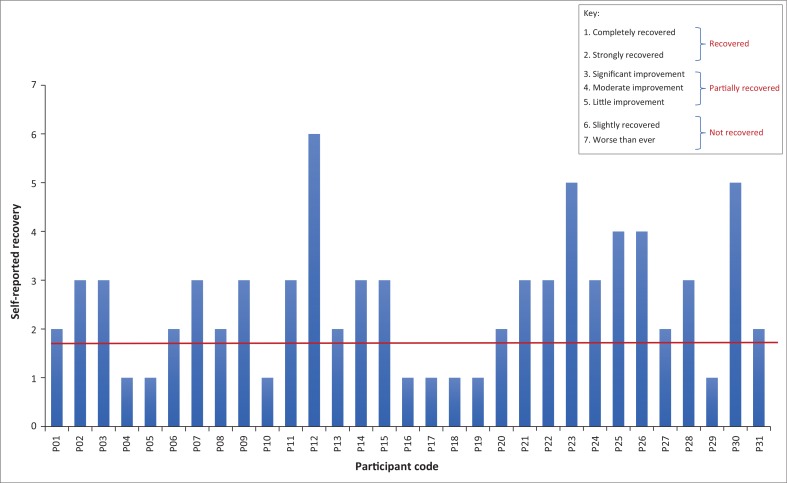
Self-reported recovery for all participants on a 7-point Likert scale at 6 months post-intervention.

As seen in Figure 1, self-reported long-term recovery was measured on a 7-point Likert scale at the 6-month follow-up and ranged from fully recovered to worse than before (Van Linschoten et al. 2009). Patients were classified as having been ‘recovered’ if they thought they had recovered well or had recovered completely (scores of 1 and 2). The patients who indicated that they felt worse or had only minimally recovered were classified as ‘not recovered’ and had scores of 6 or 7 (Rathleff et al. 2015; Van Linschoten et al. 2009). Only one participant (P12, female, 17 years-old) was ‘not recovered’, while half the others were ‘fully recovered’ and half were ‘partially recovered’ with scores ranging between 3 and 5.

When asked retrospectively to describe why they had initially chosen to participate in the study, half of the participants had the goal of returning to sport or a specific activity after the intervention. The patients’ expectations prior to participation are summarised in Table 2.

**TABLE 2:** Percentage of respondents who identified different factors as their expectations or goals of treatment at the time that they volunteered for the study.

Variable	Pain-relief	Education (understanding causes of knee pain)	Return to sport or activity	Sport performance goals	Self-management (learning exercises and strategies to prevent pain)
Number of respondents	9	7	15	2	5
Percentage (%)	29	22.6	48.4	6.5	16.1

Category I: Patients expectations of a 6-week physiotherapy intervention.

‘I wanted to go back to competing for my university cross-country team.’ (P19, female, 24 years-old)

‘I am an active person and my pain impairs my functioning and enjoyment of life.’ (P26, female, 31 years-old)

About a third of participants were mainly concerned with pain relief (29%).

‘I wanted to finally be relieved of this ongoing pain.’ (P10, female, 18 years-old)

Twenty-six per cent of participants wanted to be educated regarding the cause of their ongoing pain.

‘I want to understand the cause of the injury and how to prevent it from occurring in the future.’ (P16, female, 40 years-old)

Sixteen percent of the participants wanted to learn appropriate exercises that would allow for appropriate self-management of the condition.

‘I want to learn better training and preparation strategies for running and how to stretch better.’ (P20, female, 29 years-old)

A small percentage (6.5%), were already active but wanted to reach specific sport performance goals.

‘Although I was new to running, I had a dream to run a marathon. I could not achieve this with the knee pain I was experiencing.’ (P03, female, 39 years-old)

Most of the participants (80.6%) reported that at least one of their main expectations or goals had been met 6 months after the intervention period. Patients’ perceptions of recovery following the intervention are summarised in Table 3.

**TABLE 3:** Percentage of respondents who identified that the treatment had or had not met their expectations.

Variable	At least one expectation/ goal of intervention was achieved	At least one expectation/goal of intervention was partially achieved	Expectation of intervention not achieved	Secondary problems arose after the study period
Number of respondents	25	10	2	3
Percentage (%)	80.6	32.3	6.5	-

Category II: Patients perceptions of recovery following a 6-week physiotherapy intervention.

‘I ran my first half marathon in February 2017 and it was the first time that I could run without experiencing pain in my knee.’ (P24, female, 40 years-old)

One-third (32.3%) described one of their main expectations or goals as partially or somewhat achieved.

‘I have gone back to mountain biking, but not ballroom dancing.’ (P26, female, 31 years-old)

Two participants (6.5%) did not feel that they had recovered or that their expectations had been met six months after intervention.

‘The pain with horse-riding is the same.’ (P13, female, 14 years-old)

Three participants (9.7%) mentioned that although their knee pain had subsided they were struggling with other injuries, such as iliotibial band syndrome, foot pain and Achilles tendon pain. The pain had developed on the opposite side to the knee pain in all three cases.

‘My knee is no longer painful, but I have now developed an Achilles tendon problem on my left leg (opposite side to the knee pain).’ (P14, female, 39 years-old)

### Patients’ perceptions on the effect of the intervention on their ability to perform activities of daily livings six months after a physiotherapy intervention

The final category that was identified was patients’ descriptions of the activities that were easier or more difficult following the intervention. Twenty-nine participants (93.5%) reported improvements in activities of daily living that they previously found difficult. These activities included sitting with legs crossed, stair climbing, running, jumping, squatting, weight training, cycling, standing, walking and lunging. Of these, 10 participants (32.3%) reported that all activities were easier.

However, 13 (42%) patients reported that they were still struggling with an activity that they had previously found difficult. These activities include squatting, lunging, weight lifting, prolonged sitting, jumping kneeling, stair climbing and squash.

Two patients reported that their fear avoidance had been addressed and that they were no longer afraid to do certain activities.

‘In the past I was very afraid to perform any sudden or fast movements. This is now easier, and my knee is more stable.’ (P02, female, 18 years-old)

## Discussion

### Outline of the results

A major challenge in the treatment of PFP is that participants tend to improve with exercise but don’t recover fully (Van Linschoten et al. 2009). The 6-month follow-up showed that half of the participants (48.6%) recovered fully and half reported being partially recovered 6 months after an exercise intervention. This is similar to findings from a previous exercise intervention study that found that 43% had recovered at 3-month follow-up and 62% at 12-month follow-up (Van Linschoten et al. 2009). However, this means that the other half improved, but did not recover fully. Therefore, future research needs to establish the factors that are preventing a full recovery and strategies to prevent reoccurrence. In our study, one of these factors could be whether or not participants continued self-management after the 6-week supervised exercise period, as this was not measured.

Many patients identified returning to a specific sport or activity as the main reason for seeking treatment. This implies that the pain had inhibited their participation in these activities. Given that the participants had all experienced PFP for more than 3 months at the time of recruitment, it is possible that central mechanisms, located in the brain and spinal cord may have contributed to the ongoing pain and movement dysfunction (Rathleff et al. 2013). When musculoskeletal pain starts to become chronic the influence of affective and cognitive factors should be considered as described in the fear-avoidance model of pain.

The fear-avoidance model describes physiological factors that increase the risk of disuse and avoidance behaviours, thereby resulting in disability and chronicity of symptoms (George & Stryker 2011). Changes in fear-avoidance behaviour have been suggested as an important predictor of functional outcome in patients with PFP (Piva et al. 2009). Specifically, kinesiophobia and catastrophising behaviours have been thought to result in increased pain, disuse and disability in patients with PFP (Doménech, Sanchis-Alfonso & Espejo 2013). Therefore, addressing these maladaptive beliefs and behaviours and encouraging return to activity should be considered important components of treatment in PFP.

Two patients described that they were ‘no longer afraid’ to do certain activities and these same two patients (P19 and P06) reported that they had ‘recovered fully’ at 6 months after intervention as measured on the 7-point Likert scale. Addressing fear-avoidance behaviours using a cognitive behavioural approach is an important component of treatment and may influence patients’ long-term perception of recovery as they realise that these activities will not cause permanent damage to their knees (Smith et al. 2018).

Five participants wanted to learn exercises to self-manage their pain. Promoting self-efficacy is an important component of rehabilitation programmes for chronic musculoskeletal pain (Miles et al. 2011). This helps patients to take ownership of their condition and treatment and avoid over-reliance on experts (Rathleff et al. 2017).

Education may promote self-efficacy and help to address fear avoidance (Barton & Rathleff 2016). Twenty-two participants identified education regarding understanding the cause of their pain as an important expectation of treatment. According to the self-determination theory (SDT), a patient’s autonomous motivation may improve adherence to treatment (McLean et al. 2010). Autonomous motivation is the perception of valued benefits and the willingness to participate in treatment (Lonsdale et al. 2012). Accurately informing patients about their condition and treatment options has the potential to empower patients and optimise their care, provided that the information is based on the best available evidence, and that it addresses the needs and preferences of the individual (Barton, Holden & Rathleff 2018).

Three patients reported that they were experiencing secondary injuries following the intervention period, despite improvements in knee symptoms. It has been suggested that biomechanical approaches to treatment could, in some cases, result in a carry-over effect to other locations if patients overcompensate on certain features (Smith et al. 2018). Clinicians should be aware of this and treat the patient holistically rather than just focusing on structural and biomechanical abnormalities as this could have negative implications.

### Practical implications

Patients’ expectations of treatment need to be specifically elicited and addressed by clinicians. A focus on addressing functional ability and getting patients back to activities that add value to their lives is more important than complete resolution of pain, especially in chronic cases where multiple factors may be contributing to the ongoing pain (Medina-Mirapeix et al. 2009). Good patient-physiotherapist communication is essential to encourage adherence to treatment, especially in the case of chronic pain. Physiotherapists need to demonstrate warm and empathic communication and to assist the patient in cultivating positive expectations as this may decrease patient anxiety and improve adherence (Lonsdale et al. 2012).

Objective measures of recovery are important; however, clinicians should not neglect subjective opinions of how patients are feeling about their recovery. It is important to establish whether the patient feels that they have progressed in their recovery, regardless of whether their objective measures such as strength and motional analysis findings have shown improvements. Physiotherapists need to identify which measurable outcomes are most meaningful to the patient.

### Limitations of the study

There are several limitations related to the chosen study design and the approach used. A limitation of the retrospective nature of the qualitative interviews is the extent to which participants remember their feelings at the onset of the study (Ashby & Schoon 2012). The addition of an introductory interview at the onset of the study might have provided more accurate information about the patients’ expectations and beliefs at the time. In addition, no member checking was conducted with the participants to ensure that the information provided had been accurately reported prior to the analysis.

The telephonic mode in which the interviews were conducted has limitations. Although patients might feel more comfortable with the increased anonymity in telephonic interviews; it deprives the interviewer of seeing the participants’ non-verbal cues and body language (Sturges & Hanrahan 2004). In addition, interviews were not recorded; therefore, the process relied on accurate note-taking by the interviewer. Fortunately, note-taking is easier telephonically than face-to-face, as research has shown that note-taking is a distraction during face-to-face interviews (Sturges & Hanrahan 2004). Participant bias might have been introduced in the telephonic interviews as the interviewers (M.M. and D.L.) had also administered the interventions. Therefore, a therapeutic relationship had been established and this might have influenced the participants’ responses.

The participants had previously received a biomechanical exercise intervention as exit interventions were part of this larger study. All of the included participants presented with biomechanical contributing factors on initial assessment and the sample mainly consisted of active and working individuals. It is therefore unclear if these findings can be extrapolated to populations with different functional goals, and if including a broader range of participants could alter the findings in future research.

We have identified expectations that could be generalised to other participants; however, how these factors influence recovery and prognosis remains unknown. Our study showed that the majority of patients improved in terms of function and self-reported recovery at a 6-month follow-up. However, it is unclear how this compares to other exercise interventions and if the effects would be maintained at 12 months or even a few years later. These limitations should be addressed in future research.

### Recommendations

The treatment of PFP is challenging and complex, given its propensity to become chronic. PFP is a multifactorial condition with many possible contributing factors (Davis & Powers 2010). In our study, the average duration of symptoms was over a year (16.5 months), indicating that symptoms were chronic in most of the participants. The physical and psychological effects of chronic PFP can be additional barriers to recovery (Sanchis-Alfonso et al. 2016). Future research should include individually tailored treatments that holistically encompass any physical, biological, psychological and social factors (Falla & Hodges 2017). A long-term follow-up is essential in PFP to ensure that treatment effects are maintained. To reduce pain in the long term, exercise interventions need to prevent reoccurrence; therefore, one needs to ascertain what inhibits full recovery. This should be addressed in future research.

Therapists’ communication behaviours need to support patients’ psychological needs and motivate them to change their health-related behaviours and accept responsibility for their health in a non-judgemental way (Lonsdale et al. 2012). Interventions should be tailored to individuals’ reasons for non-adherence and this requires an understanding of patients’ experiences, priorities and views. Their beliefs should be respected and validated, and therapists should collaborate with the patients and involve them in decision-making in order to empower them (Butow & Sharpe 2013). Our study suggests that patients’ expectations of treatment and perceptions of recovery after treatment may influence prognosis, but this needs to be confirmed with future research on a larger sample.

## Conclusion

The diagnosis and causal mechanisms of PFP are not well understood and, therefore, the long-term prognosis tends to be poor. Most of the research on PFP to date has focused on quantitative, objective studies, where patients’ subjective experiences are neglected. As the condition tends to become chronic, fear-avoidance behaviours, and barriers to adherence should be included in treatment. Clinicians should consider each patient’s goals, address expectations and facilitate patients to take responsibility for their recovery in order to achieve better outcomes. Future research on individualised treatment that includes addressing psychological contributing factors should be investigated as these need to be included in the holistic management of this condition.
